# Integrated analysis reveals the regulatory mechanism of the neddylation inhibitor MLN4924 on the metabolic dysregulation in rabbit granulosa cells

**DOI:** 10.1186/s12864-024-10118-3

**Published:** 2024-03-06

**Authors:** Mengjuan Chen, Yuqing Liu, Mingzhong Zuo, Meina Zhang, Zhitong Wang, Xin Li, Dongdong Yuan, Huifen Xu, Guangqing Yu, Ming Li

**Affiliations:** College of Animal Science and Technology, Henan Agricultural University, Zhengzhou, 450046 P. R. China

**Keywords:** Rabbit, MLN4924, Neddylation, Granulosa cells, Transcriptome, Metabolome

## Abstract

**Background:**

Neddylation, an important post-translational modification (PTM) of proteins, plays a crucial role in follicular development. MLN4924 is a small-molecule inhibitor of the neddylation-activating enzyme (NAE) that regulates various biological processes. However, the regulatory mechanisms of neddylation in rabbit ovarian cells have not been emphasized. Here, the transcriptome and metabolome profiles in granulosa cells (GCs) treated with MLN4924 were utilized to identify differentially expressed genes, followed by pathway analysis to precisely define the altered metabolisms.

**Results:**

The results showed that 563 upregulated and 910 downregulated differentially expressed genes (DEGs) were mainly enriched in pathways related to cancer, cell cycle, PI3K-AKT, progesterone-mediated oocyte maturation, and PPAR signaling pathway. Furthermore, we characterized that MLN4924 inhibits PPAR-mediated lipid metabolism, and disrupts the cell cycle by promoting the apoptosis and proliferation of GCs. Importantly, we found the reduction of several metabolites in the MLN4924 treated GCs, including glycerophosphocholine, arachidic acid, and palmitic acid, which was consistent with the deregulation of PPAR signaling pathways. Furthermore, the increased metabolites included 6-Deoxy-6-sulfo-D-glucono-1,5-lactone and N-Acetyl-D-glucosaminyldiphosphodolichol. Combined with transcriptome data analyses, we identified genes that strongly correlate with metabolic dysregulation, particularly those related to glucose and lipid metabolism. Therefore, neddylation inhibition may disrupt the energy metabolism of GCs.

**Conclusions:**

These results provide a foundation for in-depth research into the role and molecular mechanism of neddylation in ovary development.

**Supplementary Information:**

The online version contains supplementary material available at 10.1186/s12864-024-10118-3.

## Introduction

In mammals, the ovary plays a critical role as the primary reproductive endocrine organ, while follicles serve as the functional units of the ovaries, and the quality and quantity of follicles within the ovaries are closely linked to the reproductive capacity of female animals [1]. Therefore, it is crucial to analyze the mechanism of follicle development for improving the fertility of livestock and poultry. The process of follicle formation is a complex, well-orchestrated process of synchronization between oocyte maturation and the proliferation of adjacent GCs [2]. Follicle growth and oocyte maturation are strictly regulated by the dynamic transcriptional changes in both ovarian cells and numerous extra ovarian signals [3]. GCs secrete cytokines and steroid hormones, influencing follicle development and oocyte maturation via paracrine or autocrine mechanisms [4, 5]. Dysfunction of GCs could lead to abnormal follicle development and ovulation, subsequently resulting in a decrease in reproductive capacity [6]. Previous studies reported that GCs commonly cease proliferation and undergo apoptosis in the majority (99.9%) of growing follicles, ultimately destined for atretic degeneration [7]. The balance between granulosa cell proliferation and apoptosis is also crucial in follicular dominant selection [8].

Neddylation, a ubiquitylation-like protein modification that covalently conjugates the ubiquitin-like protein, neuronal precursor cell-expressed developmentally down-regulated protein8 (NEDD8), to target substrate protein, is pivotal for the biological functions of cell proliferation, growth, senescence, autophagy, apoptosis, and protein interaction [9–11]. This process is analogous to ubiquitination, which is catalyzed by E1 NEDD8-activating enzyme (NAE), E2 NEDD8-conjugating enzyme (UBC12 or UBE2F), and E3 ligase, in which NAE is a heterodimer composed of APPBP1 (also known as NAE1) and UBA3 [12]. Recently, accumulated studies indicated the essential physiological significance of neddylation in heart development [13], embryonic development [14], synapse formation and maturation [15, 16], adipogenesis [17], and tumor development [18]. Specific knockout of Nedd8, resulted in abnormalities in ovarian development in female zebrafish, ultimately affecting the normal ovulation process [19]. In addition, the loss of the Uba3 gene that encodes the NEDD8-activating enzyme catalytic subunit led to intrauterine death in mice during implantation. Not surprised, dysregulation of neddylation will have a pronounced effect on the organism. However, the molecular mechanism of neddylation during ovary development remains largely unknown.

MLN4924 is a small molecule selective inhibitor of NAE, a crucial regulator of the Cullin Ring Ligases E3 (CRL) [20], which can specifically block the whole neddylation modification, thereby resulting in the inactivation of CRL [21], affecting a variety of biological processes [18, 22]. In recent years, several studies have demonstrated that MLN4924 has the ability to suppress lipid buildup and effectively enhance mitochondrial fatty acid oxidation (FAO) [23, 24]. After the utilization of MLN4924 to disrupt NAE activity, it arrests the development of oocytes at the MI phase, resulting in a delay in the development of embryos [25, 26]. Moreover, the inactivation of neddylation by MLN4924 induces apoptosis of granulosa cells, hindering the development of sheep granulosa cells [27]. While these studies emphasize the importance of neddylation in female germ cells, there is a paucity of data on the fertility implications of neddylation in animals, and the underlying mechanism of its effect remains unknown.

Rabbits serve as multi-purpose livestock, not only as valuable animal models for medical research but also as economically significant breeding animals and companion animals [28, 29]. Rabbit meat, known for its highly nutritious profile with low fat and cholesterol content, as well as its high protein content, enjoys widespread popularity [30]. Improving rabbit reproductive performance stands as a key strategy for increasing rabbit meat production. Thus, in our study, we selected the New Zealand white rabbit as the research model to delve deeper into the molecular mechanism of neddylation in follicle development. We applied transcriptomic and metabolomic analyses to investigate differences in gene expression and metabolism in rabbit ovarian GCs following neddylation inhibition by MLN4924. This allows us to identify impactful pathways involved in the process. Furthermore, we assessed the expression of mRNA and proteins related to the PPAR signaling pathway and cell cycle pathway in rabbit GCs. Collectively, the study unveils the molecular basis of neddylation in the regulation of GCs, further demonstrating the significance of neddylation in ovary development. The E1 inhibitor of neddylation (MLN4924) has undergone clinical trials for ovarian cancer [31, 32]. Consequently, this research not only provides insights into rabbit breeding, but may also establishes a foundation for investigating potential treatments for specific human ovarian diseases and reproductive physiology.

## Results

### Analysis of differentially expressed genes (DEGs) in GCs following treatment with DMSO and MLN4924

**Fig. 1:**
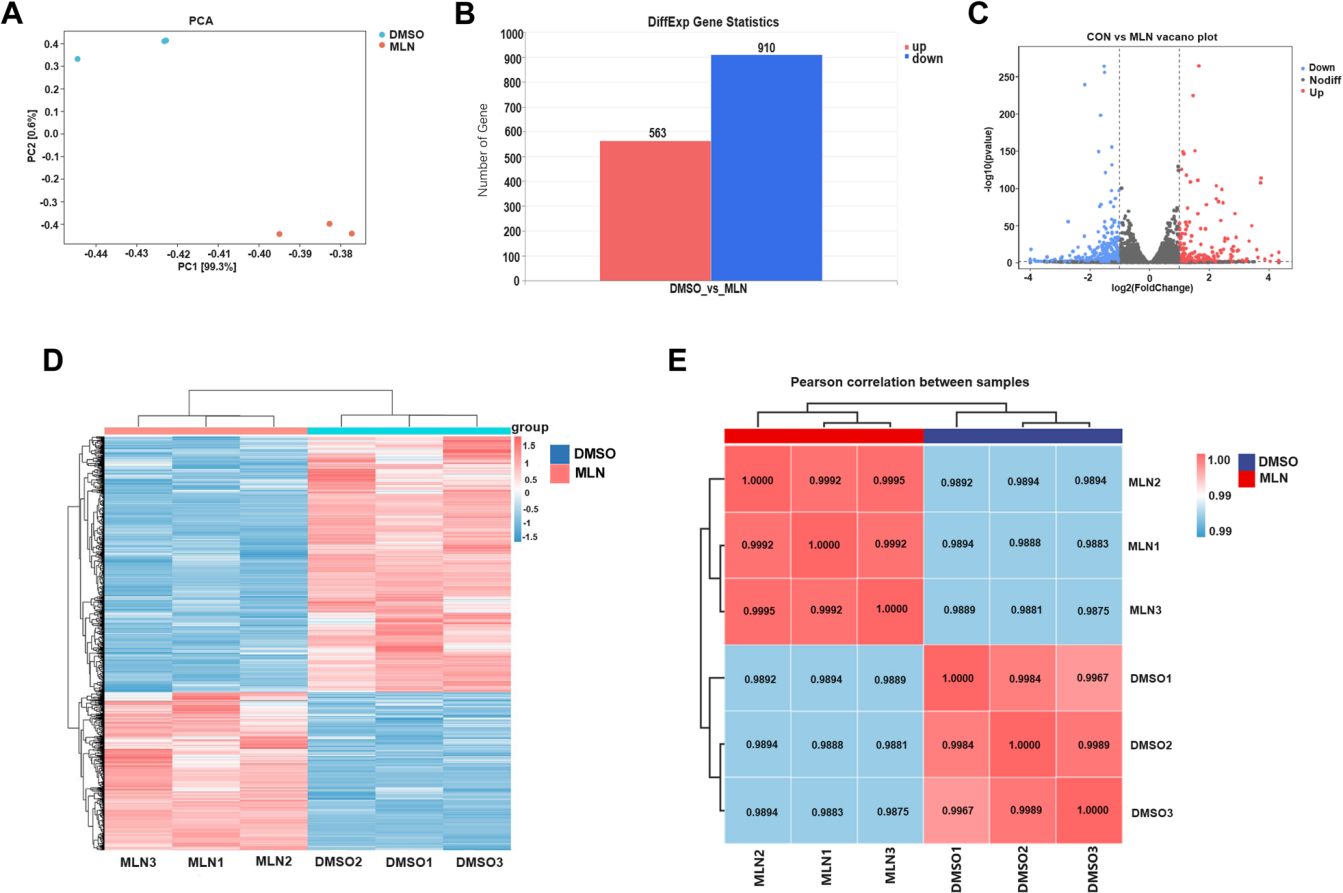
Different expression analysis in genes of New Zealand white rabbit ovarian granulosa cells treated with DMSO and MLN4924. **A** PCA score plot of transcriptomes. PC1 and PC2 coordinates represent the first and second principal components, respectively, with percentages indicating their contributions to sample variance. The control group samples are represented in blue, while the MLN4924 treatment group samples are represented in red. **B** The number of up-and down-regulated differentially expressed genes (DEGs). **C** Volcano plot for DEGs. The x-axis represents the logarithmic fold change between the two groups, and the y-axis represents the negative log10 value of the FDR for the group difference. Red points indicate up-regulated expression level in the MLN group compared to the DMSO group, blue points indicate down-regulated expression and gray points indicate no difference (criterion: log_2_FC ≥ 1.2, *P* < 0.05). **D** The heatmap shows the relative expression of DEGs. In the diagram, each column represents a sample, and each row represents a gene. Gene expression in different samples is depicted in varying colors. The redder the color, the higher the expression level, whereas the bluer the color, the lower the expression level. **E** Pearson correlation between samples. The left and upper sides show the clustering situation of the samples, while the right and lower sides of the graph show the sample names. The numbers indicate the correlation coefficient between the samples

A total of 45,436,110 and 43,644,251 raw reads were generated from the GCs samples of the CON group and MLN group, respectively. After filtering the raw reads for quality control, we obtained 44,601,646 and 42,776,095 high-quality valid reads for CON and MLN, respectively, with a Q30 base percentage of 94.89% and above (Supplemental Table S3). Valid reads were aligned with the rabbit reference genome, with a comparison efficiency ranging from 94.39 to 94.60% (Supplemental Table S3). The PCA score plot indicated scattered samples between groups and clustered samples within groups, reflecting good repetition within the groups and significant differences between the groups (Fig. 1A). Following MLN4924 treatment, 1473 differentially expressed genes (DEGs) were identified (*P* < 0.05, |log2 FC| >1.2), including 563 upregulated DEGs and 910 downregulated DEGs (Fig. 1B-D). The Pearson correlation matrix result was consistent with the results of PCA analysis, showing high similarity among biological replicates (Fig. 1E). The top 10 DEGs, including *MMP10*, *OTOGL*, *KLHL14*, *CSF3*, *KATNAL2*, *MAK*, *HKDC1*, *KCNJ16*, *GRM2*, *NR1H4*, were listed in Supplemental Table S4.

### Functional enrichment and analysis of differentially expressed genes (DEGs) in GCs treated with DMSO and MLN4924

**Fig. 2:**
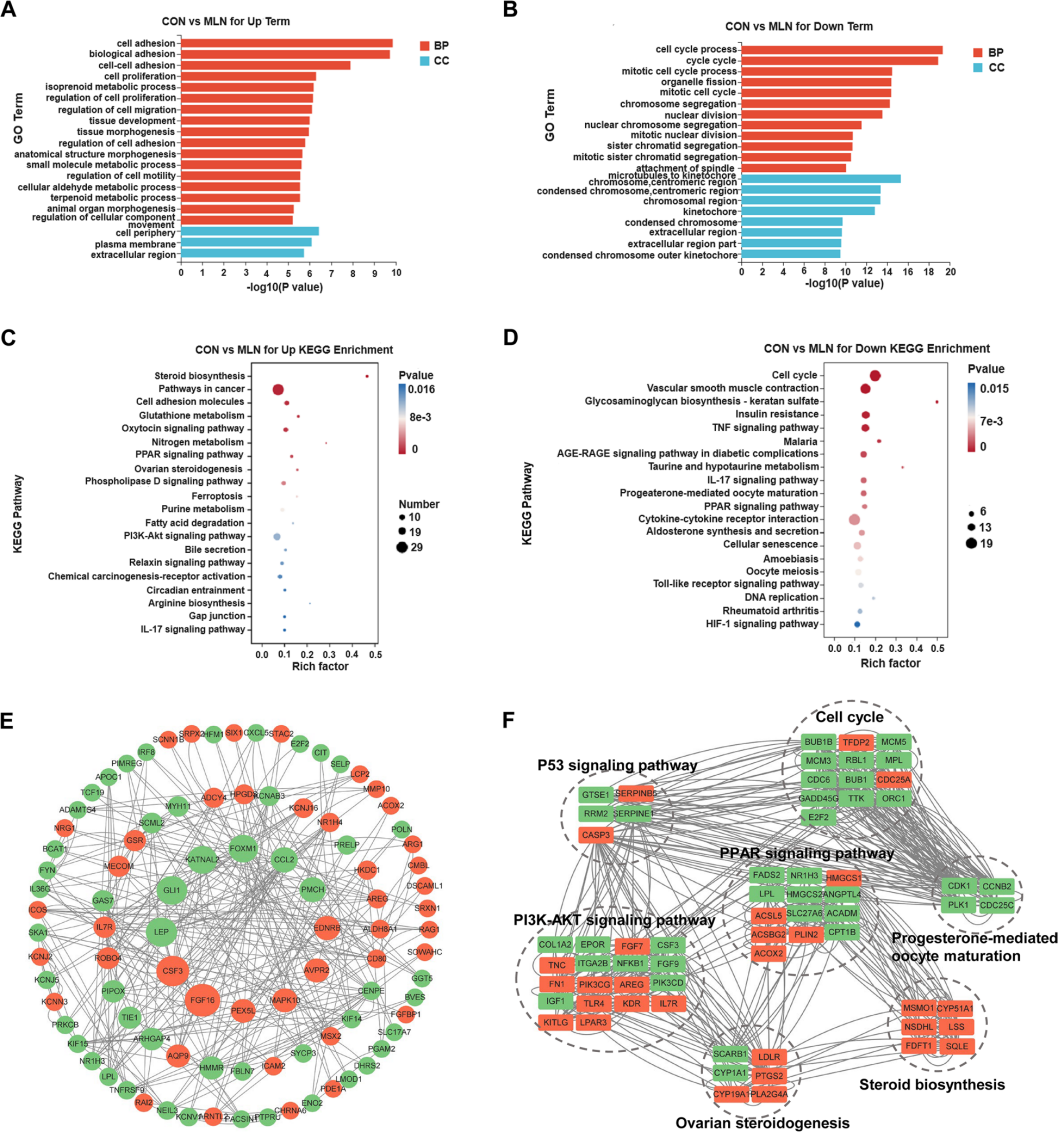
Functional enrichment analysis of DEGs in GCs of New Zealand white rabbits treated with DMSO and MLN4924. **A** Significantly up-regulated genes were examined using GO pathway enrichment (*P* < 0.05) histogram statistical analysis. **B** Significantly down-regulated genes were examined using GO pathway enrichment (*P* < 0.05) histogram statistical analysis. The x-axis represents the GO Term, while the y-axis represents the -log10 (*P*-value) enrichment of the GO Term. The orange color indicates a biological process, and the blue color indicates cellular component. **C** Scatter plot statistical analysis of significantly up-regulated genes using KEGG pathway enrichment (*P* < 0.05). **D** Significantly down-regulated genes were examined by using KEGG pathway enrichment (*P* < 0.05) scatter plot statistical analysis. The size of the point represents the number of enriched genes, and the color intensity indicates the significance level (the redder the color, the smaller the *P* value). **E** PPI analysis of DEGs using Cytoscape software. Orange circles represent up-regulated genes, and green circles represent down-regulated genes, arranged by betweenness centrality. The greater the centrality of the number, the larger the circle. **F** Interaction networks of differentially expressed proteins in major pathways significantly altered by MLN4924 treatment. Orange nodes indicate upregulated proteins and green nodes indicate downregulated proteins

To further investigate the functions of DEGs, enrichment analysis was conducted separately for upregulated and downregulated genes. The top 20 significantly enriched Gene Ontology (GO) and Kyoto Encyclopedia of Genes and Genomes (KEGG) pathways in each group were selected and displayed. According to GO term enrichment results, upregulated DEGs showed significant enrichment in cell adhesion, biological adhesion, cell-cell adhesion, and cell proliferation (Fig. 2A). Conversely, downregulated DEGs were substantially enriched in cell cycle process, cytokine-cytokine receptor interaction, vascular smooth muscle contraction, and TNF signaling pathway (Fig. 2B). In addition, the KEGG analysis results indicated that the upregulated DEGs were predominantly enriched in the pathways including cancer, PI3K-AKT signaling pathway, cell adhesion molecules, oxytocin signaling pathway, phospholipase D signaling pathway (Fig. 2C). On the other hand, downregulated DEGs were significantly enriched in cell cycle, vascular smooth muscle contraction, insulin resistance, TNF signaling pathway and cytokine-cytokine receptor interaction (Fig. 2D). Furthermore, these DEGs were also enriched in pathways such as PPAR signaling, ovarian steroidogenesis, IL-17 signaling, progesterone-mediated oocyte maturation, cellular senescence, P53 signaling, and steroid biosynthesis (Supplemental Table S5).

Next, we performed protein interaction analysis (PPI) analysis on DEGs to enhance our understanding of protein interactions within cells, thus revealing the mechanism and regulation of biological processes. As shown in Fig. 2E, most of the differential genes had significant protein-protein interaction, especially *FGF16* (fibroblast growth factor16), *CSF3* (colony-stimulating factor), *LEP* (leptin), *GLI1* (glioma-associated oncogene homolog 1), *KATNAL2* (katanin catalytic subunit A1 like 2). Subsequently, we performed PPI analysis on some of the signaling pathways of interest, as depicted in Fig. 2F. Notably, interactions among gene products related to the cell cycle and progesterone-mediated oocyte maturation were prominent. Additionally, PPAR signaling exhibited interactions with genes in other pathways. In summary, neddylation plays a crucial role in multiple intracellular signaling pathways, and the collective interactions influence GCs.

### Validation of gene expression profiles by RT-qPCR

**Fig. 3:**
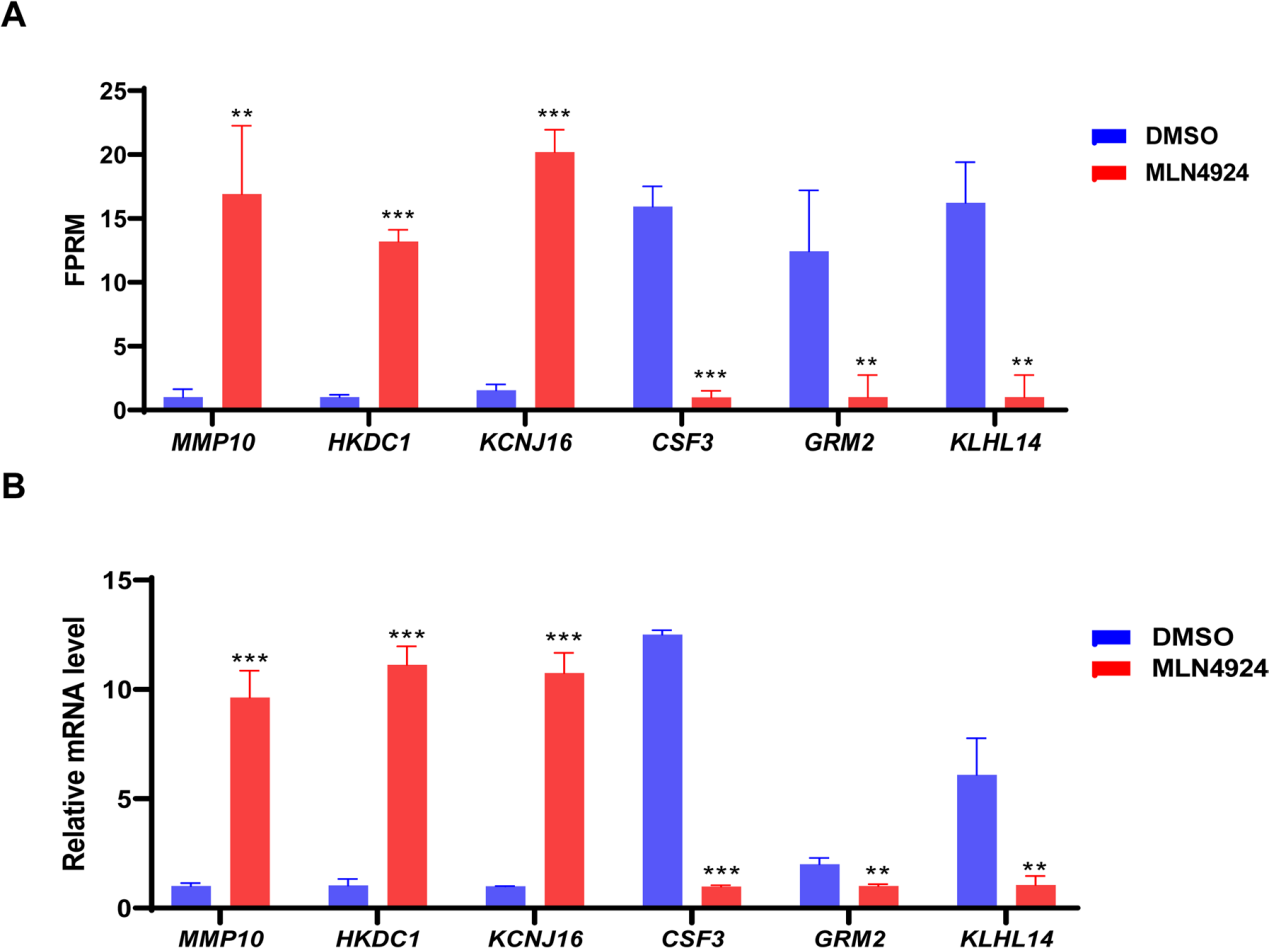
Verification of differentially expressed genes. GCs were seeded in 12-well plates and treated with DMSO or MLN4924 (1 µM) for 24 h. Cells were harvested, and gene expression was analyzed using RT-qPCR. β-actin served as a standardized internal control. *N* = 3 for each group. **A** Gene change levels based on RNA-Seq data. **B** The mRNA relative expression levels were determined by RT-qPCR. Student’s *t* test was employed to compare the differences between groups. The error bars represent mean values ± SDs. Statistical analysis results were visualized using GraphPad Prism5. *** indicates *P* < 0.001, ** indicates *P* < 0.01, * indicates *P* < 0.05

To ensure the accuracy and reliability of transcriptome sequencing data, six DEGs were randomly selected and verified by RT-qPCR. The results demonstrated that the expression levels of the *MMP10*, *HKDC1*, and *KCNJ16* genes were higher in the MLN4924 group compared to the DMSO group, while the expression levels of the *CSF3*, *KLHL14*, and *GRM2* genes were lower in the MLN4924 group than in the DMSO group (Fig. 3A). Overall, all six selected genes exhibit a consistent expression trend with the RNA-seq data, providing further confirmation of the reliability of the transcriptome data (Fig. 3A and B).

### MLN4924 suppresses the PPAR signaling pathway in GCs

**Fig. 4:**
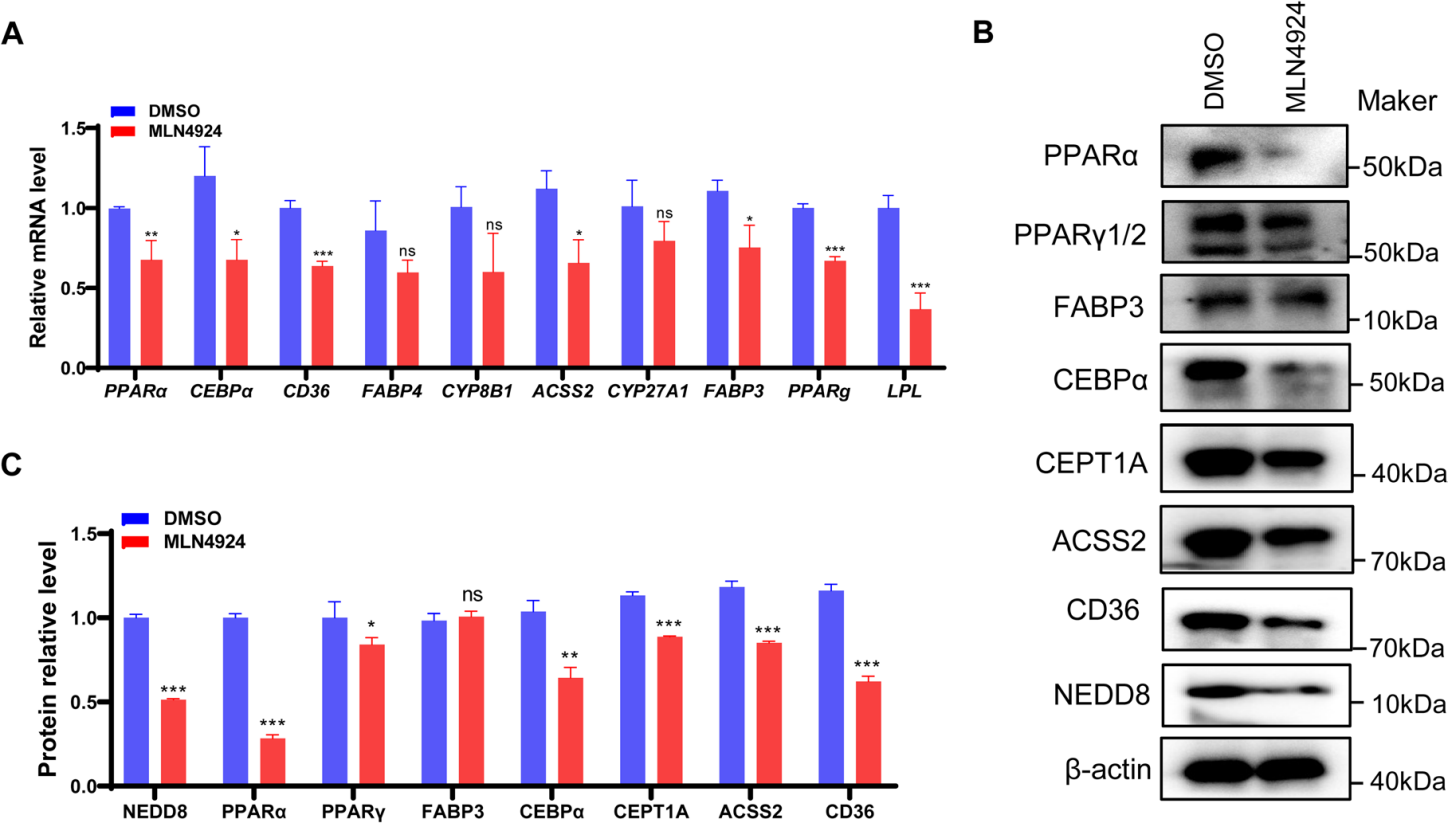
Inhibition of neddylation negatively regulates the PPAR signal pathway. DMSO and MLN4924 were added to the granulosa cell culture medium at a concentration of 1 µM. After 24 h of incubation, the cells were collected, and both RNA and protein were subsequently extracted separately (*N* = 3 for each group). **A** RT-qPCR results illustrate changes in the transcriptional levels of genes downstream of the PPAR signal pathway and genes related to lipid synthesis. **B** Western blot analysis was used to assess the protein levels of target genes of the PPAR signaling pathway and lipid synthesis-related genes. PPARg consists of two subtypes, PPARγ1 and PPARγ2. The intensities of protein bands were quantified using Image J **C**. β-actin served as the internal control. All the experiments were replicated at least 3 times. Student’s *t* test was employed to compare the differences between groups. Data are presented as mean ± SDs, and the statistical analysis results were visualized using GraphPad Prism5. *** represents *P* < 0.001, ** represents *P* < 0.01, * represents *P* < 0.05, and ns represents non-significant

As the PPAR signaling pathway was identified as the most significantly enriched pathway (Supplemental Table S5), we examined its activity changes in the two groups. Expression levels of PPAR signaling pathway-related genes were assessed using RT-qPCR and western blot analysis. As shown in Fig. 4A, the MLN4924 treatment of GCs reduced the expression of lipid synthesis marker genes *PPARA*, *PPARG*, *AC*SS2, and *CEBPA*. Simultaneously, the expression of lipid metabolism marker genes *FABP3*, *FABP4*, *CD36*, *LPL*, *CYP8B1*, and *CYP27A1* were down-regulated. The significant reduction in the protein expression level of NEDD8 after MLN4924 treatment confirmed its potent inhibitory effect (Fig. 4B and C). Moreover, the protein expression levels of PPARA, PPARγ, CEBPA, CEPT1A, CD36, and ACSS2 were markedly decreased (Fig. 4B and C). Collectively, MLN4924 treatment inhibits the differentiation and lipid metabolism of rabbit GCs.

### MLN4924 affects cell fate in GCs

**Fig. 5:**
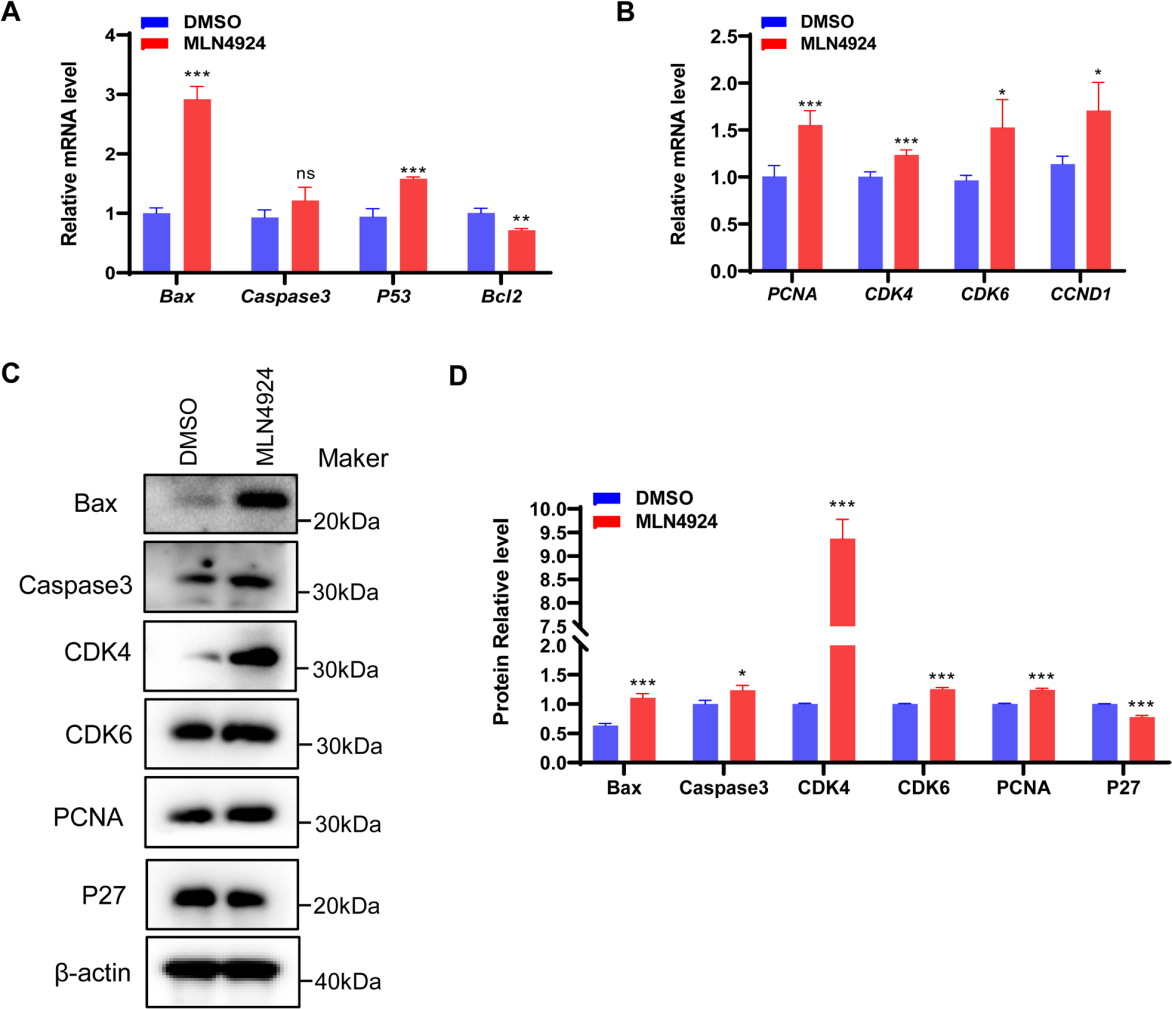
Inhibition of neddylation enhances cell cycle signaling pathway. DMSO and MLN4924 were introduced into the granulosa cell culture medium at a concentration of 1 µM. After a 24 h incubation period, the cells were collected for further analysis. Subsequently, RNA and protein of the cells were extracted respectively. **A** Changes in the expression of apoptosis marker genes *Bax*, *Caspase3*, *p53* and *Bcl2* in GCs after MLN4924 treatment, *N* = 3 for each group. **B** Changes in the expression of proliferation marker genes (*PCNA*, *CDK4*, *CDK6*, and *CCND1*) in GCs after MLN4924 treatment. **C** The protein levels of cell apoptosis and cell proliferation maker genes were assessed using Western blot analysis. **D** Quantitative results of protein expression. The intensities of protein bands were quantified using Image J. β-actin was used as the internal control. All the experiments were repeated at least 3 times. Student’s *t* test were used to compare the differences between groups. Data are shown as mean ± SDs, and the statistical analysis results were visualized using GraphPad Prism5. *** represents *P* < 0.001, ** represents *P* < 0.01, * represents *P* < 0.05, and ns represents non-significant

To investigate the effect of MLN4924 treatment on the cell cycle of the GCs, we examined the transcription and protein levels of genes involved in cell proliferation and apoptosis. As indicated in Fig. 5A, MLN4924 treatment significantly increased the expression of pro-apoptotic marker genes *Bax*, *Caspase3*, and *P53*, while decreasing the expression of the anti-apoptotic marker gene *Bcl2*, as compared to the DMSO group. In addition, neddylation inhibition significantly promoted the expression of proliferation-maker genes *CDK4*, *CDK6*, *PCNA*, and *CCND1* (Fig. 5B). At the protein level, the apoptosis maker proteins Bax and Caspase3 were upregulated, along with the proliferation marker proteins CDK4, CDK6, PCNA, and P27 (Fig. 5C-D). These findings suggest that MLN4924 treatment regulates the cell cycle, possibly by facilitating the proliferation of rabbit GCs, consistently with CCK8 assays results (Fig. S1). Therefore, our results indicate that inhibition of neddylation disrupts the cell cycle of rabbit GCs.

### Distinctive metabolism between MLN4924-treated and untreated in GCs

**Fig. 6:**
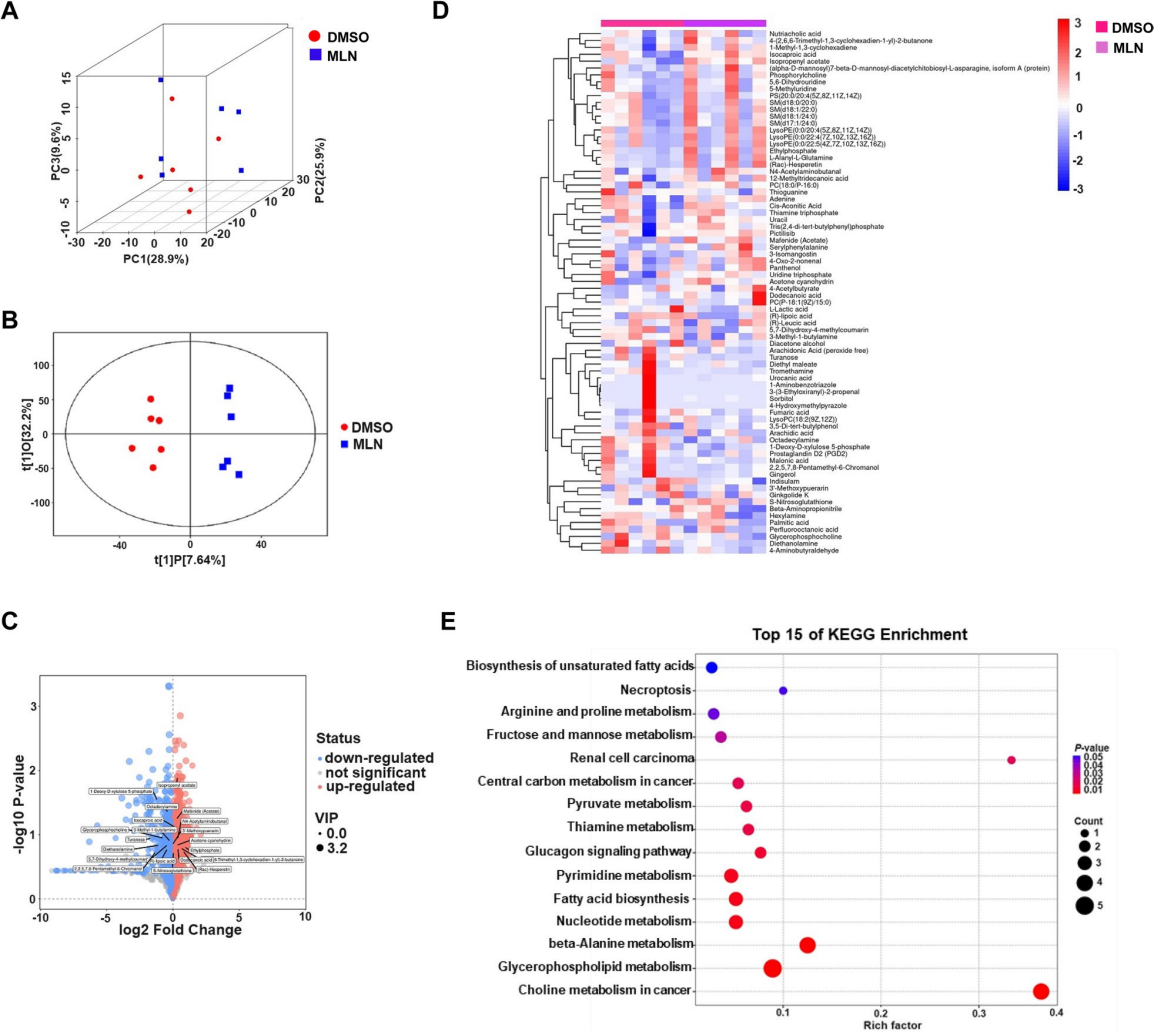
Metabolome analysis of New Zealand white rabbit ovarian granulosa cells treated with DMSO and MLN4924. **A** PCA score plot of the metabolome. **B** OPLS-DA score plot of the metabolites in GCs. The red dots represent the DMSO group, while the blue squares represent the MLN4924 treatment group. **C** Volcano plot of differential metabolites. The x-axis represents the value of the log2-transformed fold change in metabolite abundance, while the y-axis represents the -log10-transformed *P*-values from the *t* test. Red dots represent DAMs with VIP ≥ 1 and *P* < 0.05, indicating upregulation; blue dots represent DAMs with VIP ≥ 1 and *P* < 0.05, indicating downregulation. The size of the dots is proportional to the VIP value of the metabolite. **D** The heatmap shows the relative expression of metabolites in the metabolic group. In the graph, each vertical column represents a sample, while each horizontal row represents a metabolite. The various colors indicate different levels of expression. **E** Significantly differential metabolisms were examined using KEGG pathway enrichment scatter plot statistical analysis. The x-axis represents the rich factor, the y-axis indicates different pathways, the color of the point is associated with the *P*-value, and the size is indicative of the enriched count

We further analyzed the metabolic changes in ovarian GCs after MLN4924 treatment using LC-MS/MS. PCA analysis demonstrated separation between metabolites of the DMSO group and MLN group in the score plot (Fig. 6A). Further OPLS-DA analysis also indicated that there were apparent distinctions between the two groups (Fig. 6B). Based on OPLS-DA results, metabolites with P-value < 0.05 and variable importance in projections (VIP > 1) were selected for differential analysis. A total of 2535 metabolites were found to be differentially expressed, with 1534 up-regulated and 1001 down-regulated (Fig. 6C and D). The up-regulated metabolites included 3-sulfopyruvate, L-Fuculose 1-phosphate, N-Acetyl-D-glucosaminyldiphosphodolichol, D-Ribose 5-phosphate, 5’-Phosphoribosylglycimide, and 6-Deoxy-6-sulfo-D-glucono-1,5-lactone, while the down-regulated metabolites included 1-Deoxy-D-xylulose 5-phosphate, octadecylamine, glycerophosphocholine, arachidic acid, and palmitic acid. Subsequently, KEGG enrichment analysis was employed to facilitate an understanding of biological mechanisms. KEGG pathway enrichment of differential metabolites revealed 15 significant pathways (Fig. 6E), including glycerophospholipid metabolism, fatty acid biosynthesis, biosynthesis of unsaturated fatty acids, and fructose and mannose metabolism. Together, MLN4924 can regulate lipid and carbohydrate metabolism by altering differential metabolite secretion and interacting with relevant pathways.

### Integrated analysis of GCs treated DMSO or MLN4924 transcriptomics and metabolomics data reveals valuable metabolite and gene signatures

**Fig. 7:**
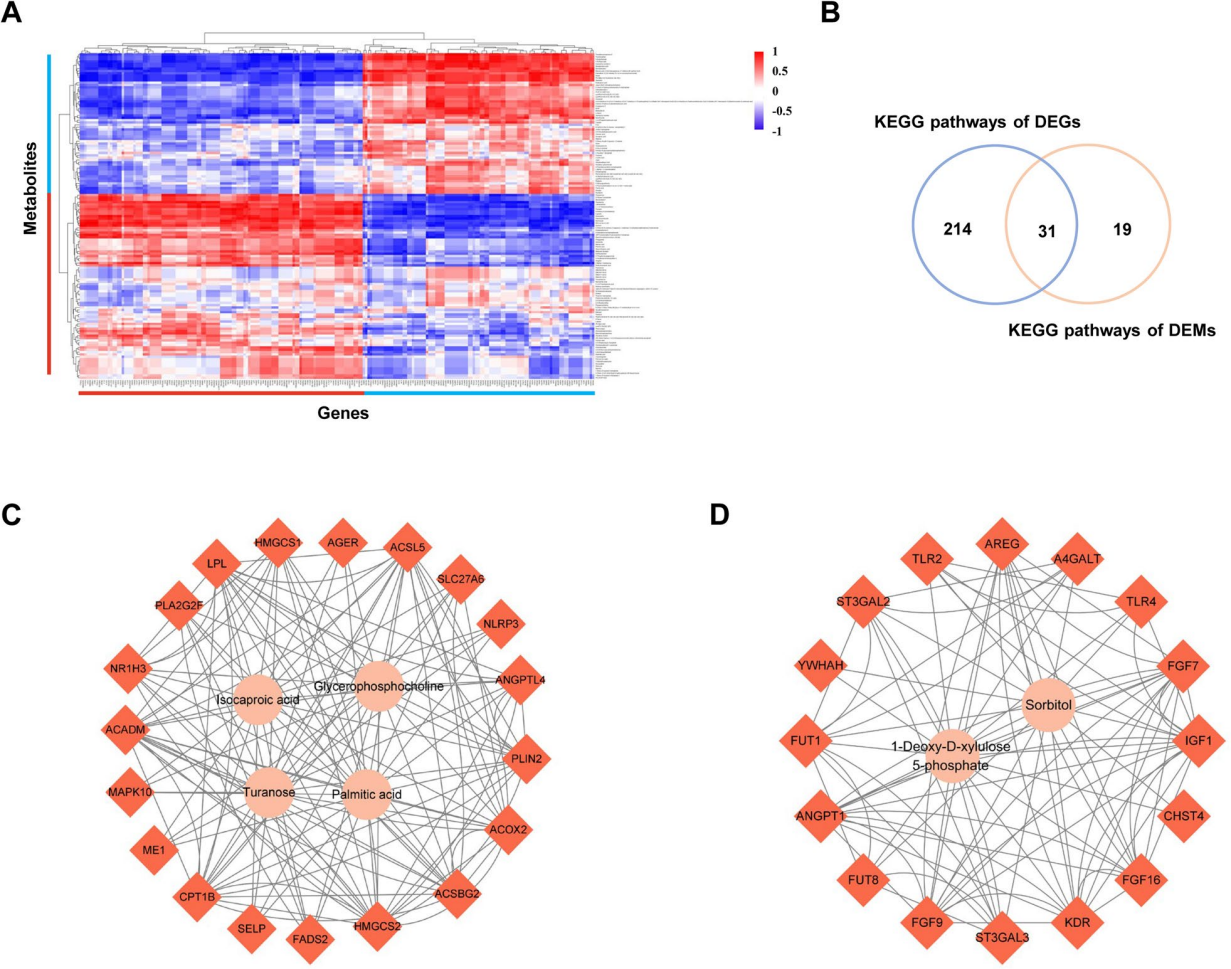
Correlated analysis of transcriptomic and metabolomic profiling of GCs from DMSO and MLN4924 treatment. **A** Heat map displaying the correlation between all DEGs and DAMs. Each row represents a differential metabolite, and each column represents a differential gene. Red indicates a positive correlation, blue indicates a negative correlation, and the darker the color, the stronger the correlation. Significant correlations are marked by an asterisk (*) to denote (*P* < 0.05). **B** Venn diagram illustrating shared KEGG terms among transcriptome and metabolome. The number in the blue circles indicates the number of KEGG signaling pathways for DEGs, the number in the orange circles indicates the number of KEGG signaling pathways for DAMs, and the overlapping sections show the number of pathways in which both omics are involved. **C** Correlation network of DEGs and DAMs related to lipids. Pink circles indicate lipid-related metabolites. Orange represents the DAMs involved in these metabolites. **D** Correlation network of DEGs and DAMs related to glucose. Pink indicates metabolites related to glucose metabolism, and orange indicates DEGs related to these metabolites

To better understand the relationships between the transcriptome and metabolome, a correlation analysis of all DEGs and DAMs was performed (Fig. 7A). Several genes exhibited significant positive or negative correlations with one or more metabolites. Subsequently, KEGG pathway enrichment results for the transcriptome and metabolome were integrated. The Venn diagram plot revealed that DEGs and DAMs shared 31 KEGG pathways (Fig. 7B). Out of these, five pathways were linked to lipid metabolism including glycerophospholipid, arachidonic acid, alpha-linolenic acid, linoleic acid metabolism and sphingolipid signaling pathway, while three pathways were associated with glucose metabolism including glycolysis/gluconeogenesis, fructose and mannose metabolism, galactose metabolism. As shown in Fig. 7C, we identified 19 genes, including *CPT1B* (carnitine palmitoyltransferase 1B), *ME1* (malic enzyme 1), *MAPK10* (mitogen-activated protein kinase 10), *ACADM* (acyl-CoA dehydrogenase medium chain), *NR1H3* (nuclear receptor subfamily 1 group H member 3), *FADS2* (fatty acid desaturase 2), and *ACOX2* (Acyl-CoA oxidase 2), that exhibit strong correlation with palmitic acid, turanose, isocaproic acid, and glycerophosphocholine. Carbohydrate plays an essential role in providing energy for follicular development. We identified 16 genes including *CHST4* (carbohydrate sulfotransferase 4), *ST3GAL3* (ST3 Beta-Galactoside Alpha-2,3-Sialyltransferase3), *FUT8* (Fucosyltransferase 8), *FUT1* (Fucosyltransferase 1) and others were highly correlated with sorbitol and 1-Deoxy-D-xylulose5-phosphate (Fig. 7D). Briefly, the results unveil that neddylation may affect the changes in intracellular lipid and carbohydrate metabolism by regulating the expression of relevant genes.

## Discussion

Reproductive performance is a critical factor influencing animal husbandry economic benefits, primarily reliant on growth and ovary follicle development. While there was increasing evidence suggesting the pivotal role of neddylation in ovary development, such as the disruption of oocyte maturation due to the loss of Nedd8 [19], its effects and underlying mechanisms on GCs development in rabbits remain unclear. In this study, we conduct a comprehensive analysis of the transcriptomic and metabolomic profiles of GCs in rabbits treated with MLN4924, revealing significant changes in gene expression and metabolites within GCs. Notably, this is the first report on the effect of neddylation inhibition on GCs using integrated transcriptomics and metabolomics analysis. These findings may provide research direction for exploring the specific mechanism by which neddylation inhibition affects the growth and development of GCs. As a result, this research may offer valuable insights and set the direction for future investigations into the intricate processes governing the impact of neddylation on GCs.

A total of 1473 DEGs were identified, and GO enrichment results showed that the differential genes were predominantly involved in biological processes such as cell adhesion, cell cycle, biological adhesion, cell proliferation, cell migration, organelle fission, nuclear division, and other biological processes. The regulation of the cell cycle encompasses various factors, such as protein post-translational modification [33, 34], lncRNAs [35–37], etc. Previous studies have highlighted the fundamental role of cell-cell adhesion in tissue and organ development [38], emphasizing the interdependence of cell cycle regulation and cell adhesion throughout each phase of the cell cycle [39]. Inactivation of neddylation has been reported to induce cell cycle arrest by regulating the G1/S transition, thereby affecting growth [40, 41]. If GCs are maintained in a state of developmental arrest, it can impact oocyte stability and normal ovulation [42]. Furthermore, follicular growth and development involve follicular wall remodeling, where the movement and migration of granulosa cells play a crucial role [43]. Significantly, we observe that the inhibition of neddylation led to a notable upregulation of these DEGs, particularly those associated with the GO term of cell migration.

The KEGG analysis revealed enrichment in pathways closely associated with growth, metabolism, and hormone production, such as PI3K-AKT, steroid biosynthesis, oxytocin, PPAR, ovarian steroidogenesis, cellular senescence, and P53, among others. Studies in recent years have underscored the crucial role of the PI3K-AKT signaling pathway in ovarian development and diseases, with its overactivation leading to premature development and apoptosis of primordial follicles [44–46]. Estrogen and progesterone, pivotal for normal reproductive function in female animals, are steroid hormones secreted by the ovary [47]. The precursors of estrogen synthesis, testosterone and androstenedione, can only be converted into estradiol and estrone by CYP19A1 when entering GCs [48]. In our research, numerous upregulated DEGs are enriched in pathways such as steroids, ovarian steroids, and oxytocin, and so it is hypothesized that MLN4924 treatment of GCs could lead to the dysregulation of steroid production. MLN4924 has been demonstrated to inhibit the expression of ER-α, a steroid hormone receptor, through SGK1-dependent cytoplasmic localization of FOXO3a [49]. PPI is composed of proteins that interact with each other, involved in every biological process that occurs within an organism [50]. Among the top ten key genes in the PPI network, six were involved in regulating cell proliferation and apoptosis, including FGF16, CSF3, GLI1, EDNRB, AVPR2, and FOXM1. MiR-144-3p has been found to block the proliferation of cells induced by high glucose by suppressing the FGF16 and MAPK signaling pathways, as reported [51]. Additionally, the knockdown of FOXM1 has been observed to enhance cell apoptosis and induce G1-phase cell cycle arrest [52]. The interconnectedness observed in the PPI network across various differential pathways in this research suggests that GCs are influenced by multiple mechanisms following MLN4924 treatment.

Lipid signaling and metabolism play a crucial role in regulating ovarian steroidogenesis and ovulation. Fatty acid (FA) β-oxidation (FAO) in oocyte mitochondria is essential for meiotic maturation, accompanied by differential expression of numerous genes involved in FAs metabolism in surrounding granulosa cells [53]. In our study, of note, the PPAR signaling pathway was the most significantly differentially expressed signaling pathway. PPARs, nuclear hormone receptors that bind to DNA, control the transcription of genes related to lipid and glucose metabolism [54]. Activation of *PPARγ* by free fatty acids or its endogenous ligands leads to the excess storage of lipids through the expression of *FASN*, *FABP4*, and *CD36* [55]. In our investigation, we examined a downregulation in the mRNA or protein expression levels of *PPARα/γ*, *CEBPα*, *FABP3*, *FABP4*, *ACSS2, CD36*, and *LPL* following exposure to MLN4924 in GCs. Similarly, Park et al. [17] found that MLN4924 downregulates these genes in fat tissues, which are involved in lipid uptake. Therefore, we speculate that MLN4924 can inhibit adipogenesis or fat accumulation in GCs by PPAR signaling, thereby influencing the normal development of follicles.

We next identified the marker genes associated with cell proliferation and apoptosis, particularly those related to the cell cycle. Our findings reveal that MLN4924 inhibition of neddylation enhances both cell proliferation and apoptosis. Recent research indicates that MLN4924 treatment promotes G2/M-phase arrest and cellular senescence by triggering a DNA damage response, leading to the accumulation of key factors such as *p53*, *Cdt1*, *p21*, *p27*, and *Wee1* [56, 57]. The apoptosis promoter *Bax*, released by mitochondria for intrinsic cellular apoptosis, showed increased expression along with *p53* following neddylation inhibition, consistent with previous studies in melanoma cell lines [58]. Moreover, in this study, the expression of the apoptosis inhibition factor, *Bcl2*, was downregulated, while the expression of *Caspase 3* was upregulated. Similar results were found in the study of sheep granulosa cells [27]. Interestingly, the examined cell proliferation marker genes exhibited significant upregulation, contrary to previous findings in cancer studies [59, 60]. This discrepancy may be attributed to compensatory proliferation triggered by apoptosis in normal tissues, warranting further investigation to elucidate the specific underlying mechanism.

Additionally, we observed significant changes in the content of some metabolites (glycerophospholipid metabolism, fatty acid biosynthesis, biosynthesis of unsaturated fatty acids, fructose and mannose metabolism, etc.) in rabbit GCs, upon the inhibition of neddylation. For instance, palmitic acid is the main fatty acid in mitochondrial fatty acid oxidation that produces ATP, excess palmitic acid can lead to lipid accumulation inside the cell [61, 62]. Arachidic acid, an unsaturated fat acid, plays a role in reducing the chance of lipid peroxidation [63]. *GDE5* can control adipocyte differentiation and lipid droplet formation by influencing the accumulation of glycerophosphocholine [64]. Importantly, the level of these metabolites reduced after MLN4924 treatment in rabbit GCs, further supporting our previous hypothesis. Inhibition of the neddylation has been demonstrated to reduce adipogenesis and lipid storage by deregulating *PPARγ* [17], which is consistent with our research findings.

Utilizing the identified differential genes and metabolites, we constructed the interaction networks and primarily observed alterations related to lipid and glucose metabolism. Balanced energy metabolism is essential for the development and maturation of oocytes. Glucose utilization and fatty acid breakdown appear to correlate in mouse COCs [65], as well as in bovine embryos [66]. Pharmacological blockage of cullin neddylation by MLN4924 (Pevonedistat) swiftly reduces hepatic glucose production and mitigates hyperglycemia in mice [67]. From the above analysis, we discover that neddylation may control the balance of energy metabolism, consequently promoting follicular development through the regulation of glucose and lipid metabolism in GCs. However, further research and assessment are needed to fully understand these intricate mechanisms.

## Conclusion

**Fig. 8:**
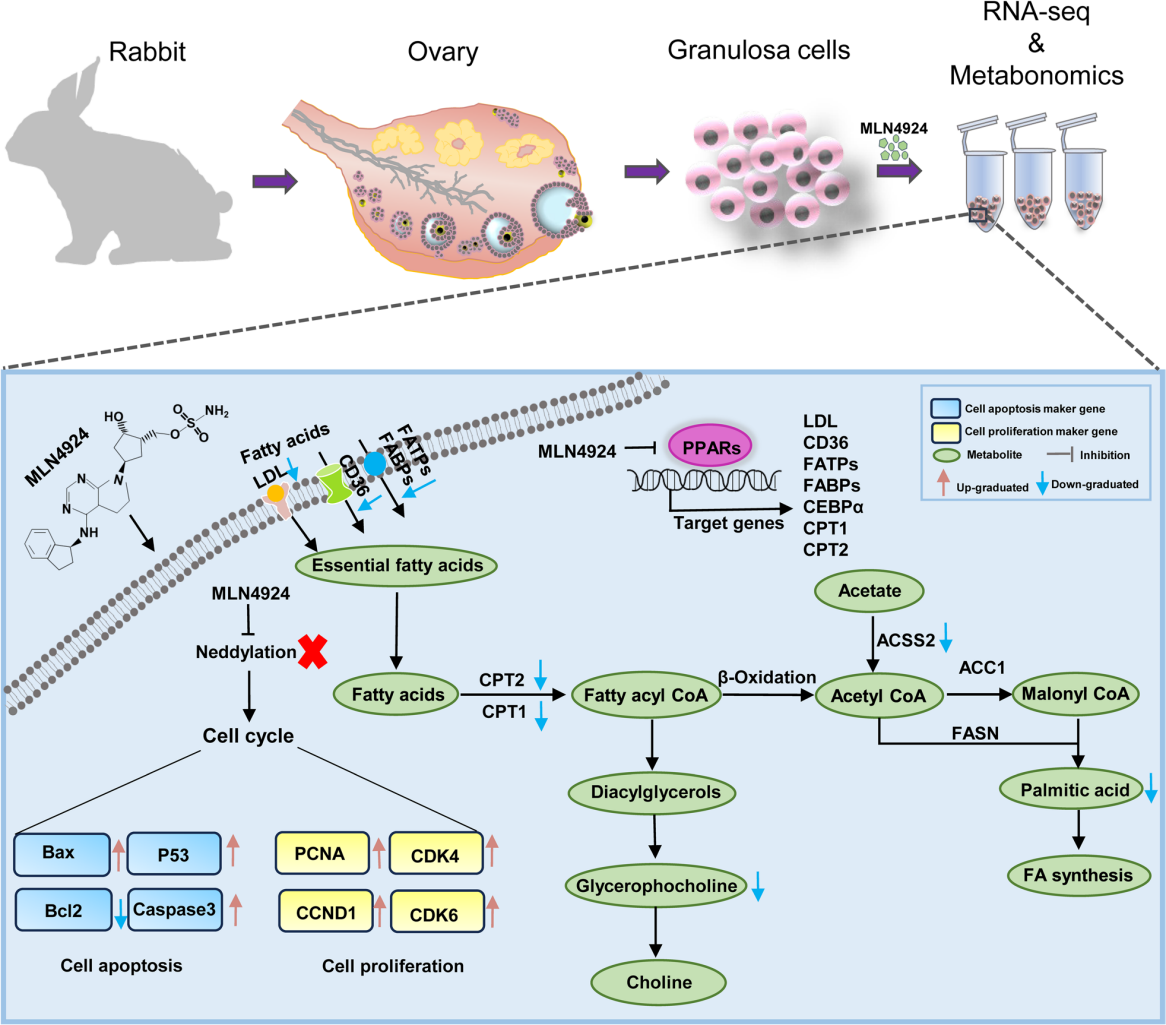
Working model of lipid synthesis induces by PPAR and cell cycle regulation in GCs after MLN4924 treatment. Rabbit follicular granulosa cells were isolated and treated with MLN4924, followed by transcriptomic and metabolomic sequencing. MLN4924 inhibits the intracellular neddylation process upon entering granulosa cells, impairing the PPAR signaling pathway, while also inducing apoptosis and cell proliferation in GCs. Inhibition of PPARα/γ down-regulates the target genes *FABP3*, *FABP4*, *CD36*, and *LPL*, which are involved in lipid transport, resulting in reduced fatty acid uptake. Similarly, the expression of *CPT1* and *CPT2* is also reduced, potentially inhibiting the process of fatty acid β-oxidation. ACSS2, responsible for linking acetate to CoA to produce acetyl-CoA, also shows reduced expression, affecting the availability of raw materials for fatty acid synthesis. Therefore, blocking neddylation within granulosa cells inhibited PPAR-mediated lipid metabolism and interfered with cell cycle progression

Our study demonstrates that MLN4924 significantly alters the expression of specific genes and metabolites in rabbit GCs. We investigated the impact on the cell cycle and PPAR signaling pathways enriched by differentially expressed genes, shedding light on the effects of MLN4924 treatment on GCs metabolism. The suppression of neddylation can lead to a significant decrease in the expression of genes involved in lipid creation and storage in the PPAR pathway, and enhance the activation of genes related to apoptosis and cell proliferation (Fig. 8). By analyzing significant enrichment pathways in both transcriptome and metabolome, we identified two metabolic pathways related to glucose and lipid metabolism. In summary, this study provides new basic data and effective information on the effect of MLN4924 treatment on rabbit GCs. Nevertheless, further experiments are required to investigate the specific molecular mechanisms through which neddylation regulates energy metabolism and to identify the targets and pathways of neddylation in regulating rabbit GCs development.

## Materials and methods

### Ethics approval statement

The experimental rabbits were sourced from Henan Chunying Biotechnology Co., Ltd. All procedures involving the killing and sampling of rabbits were conducted under strict supervision to minimize animal suffering. This study was designed and executed by the guidelines of the Institutional Animal Care and Use Committee (No. 11-0085) of the College of Animal Science and Technology, Henan Agricultural University, China.

### Experimental animals and sample collection

Twelve healthy and sexually mature female New Zealand white rabbits were chosen for the isolation of ovarian granulosa cells. The rabbit is approximately 5 months old, and its weight is maintained at 2.5 kg. To stimulate the development and maturation of female ovarian follicles, the rabbits were subcutaneous injected with 50 units perpregnant mare serum gonadotropin (PMSF) (Solarbio, China) 48 h before slaughter. Following slaughter, the ovaries were promptly placed in 37 °C PBS and then transferred to the cell chamber for the isolation of granulosa cells.

### Isolation and culture of rabbits GCs

The collected ovaries were placed in a petri dish and washed three times with PBS (phosphate-buffered saline) containing 1% penicillin/streptomycin at 37 °C. Subsequently, the ovaries were placed in a basal medium supplemented with DMEM / F12 medium (Price, China), 15% fetal bovine serum (Price, China), and 1% penicillin/streptomycin (Price, China). Follicles on the ovaries were punctured with a 1 mL syringe needle to allow the follicular fluid to flow out [68]. The collected follicular fluid was then centrifuged at 1000 rpm for 5 min, and the supernatant was discarded. The remaining fluid was incubated with basal medium at 37 °C in 5% CO_2_, with the cell medium changed every 24 h. Before experiments, GCs were seeded in 6 cm cell culture dishes at a density of 3 × 10^5^ cells per cm^2^.

### MLN4924 treatment

MLN4924 (1mM, DMSO) (MedChemExpress, USA) was dissolved in DMEM/F12 culture medium to achieve a final concentration of 1 Μm (Fig. S1A) [69]. The control group received an equivalent volume of DMSO treatment. For MLN4924 treatments, when the cell density reached 70-80%, MLN4924 (1 µM) was added into the medium and incubated for 24 h, then the cells were harvested.

### CCK8 assays

To assess cell viability using CCK-8 Cell Counting Kit (Vazyme, China), cells need to be seeded onto 96-well plates (2 × 10^3 cells per well) with complete growth medium and transfected. At 12, 24, 36, 48, and 60 h post-transfection, 10 µL of CCK-8 solution were added to each well and incubated at 37 ℃ for 2 h. The absorbance of cells at 450 nm was measured using a microplate instrument (BioTek, USA) manufactured.

### RNA extraction and sequencing

According to the manufacturer’s extraction protocol, the total RNA of cell samples was isolated using TRIzol reagent (Invitrogen, USA), and RNA quality and concentration were measured using a NanoDrop One (Thermo Fisher Scientific, USA) and agarose gel electrophoresis, respectively. The concentration of agarose was 1%. The RNA samples were stored on dry ice and sent to Shanghai Personal Bio Company (Shanghai, China) for RNA-seq. For RNA-seq, first-strand cDNA was synthesized using random oligonucleotides and Super Script II. Subsequently, second-strand cDNA synthesis was performed using DNA Polymerase I and RNase H. The resulting suspensions were treated to remove any remaining blunt ends through exonuclease/polymerase activity. Following adenylation of the 3’ end of the DNA fragment, the Illumina PE adapter oligonucleotide was attached to facilitate hybridization. To isolate cDNA fragments of the desired length (400–500 bp), the library fragment was purified using the AMPure XP system (Beckman Coulter, Beverly, CA). DNA fragments carrying linker molecules at both ends were selectively enriched through 15 cycles of PCR reaction using an Illumina PCR primer cocktail. The resulting product was subsequently purified using the AMPure XP system and quantified on an Agilent Technologies Bioanalyzer 2100 system, employing high-sensitivity DNA analysis.

Afterward, the sequencing library was then sequenced using the NovaSeq 6000 platform (Illumina, USA) and read length was 150 bp. Because the sequencing data contains some low-quality reads, we use Cutadapt (v1.15) software to filter the sequencing data to obtain high-quality sequences (clean data) for further analysis. The filtered reads were utilized to align the rabbit reference genome using HISAT2 v2.0.5.

### Analysis of gene expression data

HTSeq (0.9.1) was employed for statistical analysis, comparing the read counts of each gene as raw expression data. Subsequently, FPKM (Fragments Per Kilobase of transcript per Million mapped reads) was used to normalize the gene expression levels. DESeq (1.30.0) [70] was then used to analyze the differential expression of genes, with screening conditions set as follows: expression difference multiple |log2 FoldChange|>1.2, significant *P* value < 0.05. Simultaneously, we employed the Pheatmap (1.0.8) package in the R language to conduct a two-way clustering analysis of all the different genes present in the samples. We generated a heatmap based on the expression levels of the same gene in different samples, and the expression patterns of different genes in the same sample. The Euclidean method was employed to calculate distances, and the Complete Linkage method was used for clustering. GO (Gene Ontology) enrichment and Kyoto Encyclopedia of Genes and Genomes (KEGG) analysis [71–73] of DEGs were conducted on the Personal Cloud Platform (https://www.genescloud.cn/). The protein-protein interaction network was constructed using the STRING online tool (https://cn.string-db.org/) and visualized using the Cytoscape software.

### Metabolite extraction and LC-MS/MS analysis

The frozen samples were slowly thawed at 4 ℃ mixed with 1mL extraction solution (methanol: acetonitrile: water = 2:2:1 (V/V)), and vortexed for 30 s. The samples were frozen in liquid nitrogen for 1 min, thawed, and vortexed for 30 s, repeated this step 3 times. The samples were then sonicated in a water bath at 4 °C for 10 min and incubated at -40 °C for 1 h to precipitate the proteins. After incubation, the samples were centrifuged at 12,000 rpm (RCF = 13,800×g, *R* = 8.6 cm) at 4 °C for 15 min. The supernatant was carefully collected in an injection bottle for further detection and analysis. Quality control (QC) samples are prepared by mixing equal amounts of sample supernatant.

LC-MS/MS analysis was performed on a UHPLC system (Vanquish, Thermo Fisher Scientific), and the target compound was chromatographically separated by Waters ACQUITY UPLCBEH Amide (2.1 mm × 50 mm, 1.7 μm) liquid chromatography column. The mobile phase for the analysis consisted of two components: 25 mmol/L ammonium acetate and 25 mmol/L ammonia hydroxide in water, adjusted to a pH of 9.75 (A), and acetonitrile (B). The temperature of the automatic sampler was 4 °C and the volume of injection was 2 µL. Information-dependent acquisition (IDA) modes of mass/mass spectrometry were obtained using the orbital detector 120 mass spectrometer under the control of Acquisition Software (Xcalabur, Thermo). Raw data was converted to MZXML format using ProteoWizard and processed using R packages, followed by an internal MS2 database (BiotreeDB) for metabolite annotation. The cut-off point for comments was set to 0.3.

### Real‑time quantitative PCR (RT‑qPCR) validation

Total RNA from cultured cells was extracted with TRIzol (TransGen Biotech, China) and reverse transcribed into cDNA according to instructions (Vazyme, China). Real-time PCR Instrument (Thermo Fisher Scientific, USA) was used for RT-qPCR assay, with the reaction system established according to the manufacturer’s SYBR real-time PCR kit (Vazyme, China). The primers for RT-qPCR assays are listed in Supplemental Table S1. Based on the internal reference gene *β-actin*, the mRNA expression levels of the genes were quantitatively analyzed using the 2^−ΔΔCt^ method [74].

### Western blot analysis

Total protein from rabbit granulosa cells was extracted using RIPA (Epizyme, China) with a proteasome inhibitor. Cells were lysed as other study described previously [36]. The protein concentration was determined using the BCA kit (KeyGEN). Subsequently, the supernatants were mixed with sodium dodecyl sulfate (SDS) buffer (Epizyme, China) and boiled at 100 °C for 10 min. After separation on 10% acrylamide SDS-PAGE gel, the protein bands were transferred to the PVDF membrane (Merck Millipore Ltd, Ireland). The membranes were then blocked with 5% skim milk powder for 1 h at room temperature, followed by overnight incubation with the primary antibody at 4^◦^C. Then, the PVDF membranes were incubated with the secondary antibody at room temperature for 1 h. Finally, the blots were visualized using ECL reagent and captured with a QuickChemi 5200 imaging system (Monad Biotech, China). Antibody information is provided in Supplemental Table S2.

### Correlation analysis of differentially expressed genes (DEGs) and different accumulated metabolites (DAMs)

To investigate the potential interaction network of DEGs and DAMs, Spearman correlation analysis was used to calculate the correlation. The cor. test function tests the statistical significance of the correlation coefficient between two numerical vectors, and obtains the correlation coefficient (Corr) and the correlation *P*-value matrix. Next, the markedly distinct genes and metabolites were examined using correlation hierarchical clustering, and the Euclidean distance matrix was calculated to cluster the different metabolites and genes by the complete linkage method. The heatmap visualized the correlation between the metabolites and DEGs.

### Statistical analysis

Statistical analysis for the RT-qPCR and western blot results was performed using SPSS 26 statistical software (IBM, Armonk, NY, USA), which were presented as the means ± standard deviations (SDs). Data visualization was performed using GraphPad Prism (v 8.0) software. All experiments were repeated at least three times. Significant differences between groups were analyzed using one-way ANOVA followed by Tukey’s test (multiple groups) and Student’s *t*-test (two groups), with differences considered statistically significant if *P* < 0.05 (*P < 0.05, **P < 0.01, ***P < 0.001).

### Supplementary Information

**Supplementary Material 1.**

## Data Availability

Sequence data that support the findings of this study have been deposited in the National Center for Biotechnology Information with the primary accession code PRJNA1050663
