# Supplementary material for: Integrated analysis reveals the regulatory mechanism of the neddylation inhibitor MLN4924 on the metabolic dysregulation in rabbit granulosa cells
Source: BMC Genomics. 2024 Mar 6;25:254. doi: 10.1186/s12864-024-10118-3 (PMC10916191; doi:10.1186/s12864-024-10118-3)
Supplement: Supplementary file 1 — Supplementary Material 1. [file 12864_2024_10118_MOESM1_ESM.docx]

**

**

**Supplemental Fig. 1** CCK8 assays of MLN4924-treated GCs. **A** Cell viability of rabbit GCs treated with MLN4924 at concentrations of 0, 0.1, 0.5, 1, 5, and 10 μM, respectively. **B** Viability of rabbit GCs assessed by CCK8 assay after treatment with MLN4924 (1 μM) for 12, 24, 36, 48, and 60 h. Student's *t* test or one-way ANOVA was employed to compare the differences between groups. Data are presented as the mean ± SD (n=3). ****P*< 0.001, ***P*< 0.01, **P*< 0.05, ns, not significant.

**Supplemental Table 1. Primer information required in the experiment.**

| Primer Name | Primer Sequence (5'-3') | Length (bp) |
| --- | --- | --- |
| *MMP10*-F | CTTACCCTCTGAATGGGGCG | 177 |
| *MMP10*-R | ACTCGTCAACACCCCCATTC |  |
| *HKDC1*-F | TATTGGACATCATGGCGCGG | 186 |
| *HKDC1*-R | AGACCTGCACCTTCAAGACG |  |
| *KCNJ16*-F | AATGGCAACTGCTCGGAAGA | 148 |
| *KCNJ16*-R | AAGCTGGGCTCTCACTGTTC |  |
| *CSF3*-F | CGACTTTGCCACCACCATCT | 159 |
| *CSF3*-R | GTCAGCTCCAGGAAGCTCTG |  |
| *GRM2*-F | TCTTCCAGCCGCAGAAGAAC | 155 |
| *GRM*-R | TGTCGTCGAGTCTACCACCT |  |
| *KLHL14*-F | ATGCCATACAACAGTGCCCA | 199 |
| *KLHL14*-R | TGTCCAACCGACAGGCATAG |  |
| *Bax*-F | CCCTCCTCTCTCCTCTAGGGC | 198 |
| *Bax*-R | TGTCCAGTTCGTCGCCAATG |  |
| *Caspase3*-F | GCAAATCAATGGACTCTGGGAA | 164 |
| *Caspase3*-R | GAATGTTTCCCCGAGGTTTGC |  |
| *P53*-F | GAGTCGCAGTCGGATCTCAG | 121 |
| *P53*-R | GATCATCCACGGGAGGGTTC |  |
| *Bcl2*-F | CTTTGAGTTCGGTGGGGTCA | 152 |
| *Bcl2*-R | GTTCCACGAAGGCATCCCA |  |
| *PCNA*-F | TTTCCGCCAGTGGAGAACTG | 165 |
| *PCNA*-R | TAGGAGAAAGCGGAGTGGCT |  |
| *CDK4*-F | CAGCTACCAGATGGCGCTTA | 166 |
| *CDK4*-R | GGCTTCGGAGTTTCCACAGA |  |
| *CDK6*-F | GGAAAAATCTTGGACGTCATTGGA | 199 |
| *CDK6*-R | GGGCGCTGTAGGCAGATATT |  |
| *CCND1*-F | CTTCATTGCTCTCTGCGCCA | 190 |
| *CCND1*-R | GCAGGCTTTGAGACAATCCAC |  |
| *PPARα*-F | CGACATGGAGACGCTGTGTA | 181 |
| *PPARα*-R | CAGGTCCAAGTTTGCGAAGC |  |
| *CEBPα*-F | TGGACAAGAACAGCAACGAGT | 141 |
| *CEBPα*-R | GCAGGCGGTCATTGTCACT |  |
| *CD36*-F | ACTGGGTGAAAACAGGCACA | 184 |
| *CD36*-R | AAGGAAACCGTGTGGTCCTC |  |
| *FABP4*-F | AAGTCACCGCAGATGACAGG | 166 |
| *FABP4*-R | ATTCTGGTGGAAGTGACGCC |  |
| *CYP8B1*-F | GGGACCACCGGATGATACAC | 158 |
| *CYB8B1*-R | AAAGAGGCCATCCTCATGCC |  |
| *ACSS2*-F | GTCGAGAGAAGGGTTTCCCAG | 152 |
| *ACSS2*-R | ACGCTTGGATTTCTCTTTCAGC |  |
| *CYP27A1*-F | ACCCAGTTTGTGTTCTGCCA | 190 |
| *FABP3*-F | TTGGGGTGGAGTTCGATGAG | 154 |
| *FABP3*-R | AGCGTCAGGATGAGTTTCCC |  |
| *PPARg*-F | GCCGAGAAGGAGAAGCTGTT | 123 |
| *PPARg*-R | CAGCGGGAAGGACTTGATGT |  |
| *LPL*-F | ATGTCCACCTCCTGGGCTAT | 164 |
| *LPL*-R | GACAAAATCTGCGTCGTCCG |  |
| *β-actin*-F | ATGCAGAAGGAGATCACCGC | 148 |
| *β-actin*-R | ACTCCTGCTTGCTGATCCAC |  |

**Supplemental Table 2. Antibody information required in the experiment.**

| Antibody Name | Catalog | Source | Dilution |
| --- | --- | --- | --- |
| BAX | 2772T | Cell Signaling Technology | 1:1000 |
| Caspase3 | WL04004 | Wanleibio | 1:500 |
| CDK4 | AM8485b | abcepta | 1:1000 |
| CDK6 | D220398 | Sangon Biotech | 1:1000 |
| PCNA | AM8545b | abcepta | 1:1000 |
| P27 | WL04174 | Wanleibio | 1:500 |
| PPARα | 41359 | SAB | 1:1000 |
| PPARγ | C26H12 | Cell Signaling Technology | 1:1000 |
| FABP3 | BS7746 | Bioworld Technology | 1:1000 |
| CEBPα | 49157 | SAB | 1:1000 |
| CPT1A | 15184-1-AP | Proteintech | 1:1000 |
| ACSS2 | Ab264390 | Abcam | 1:1000 |
| CD36 | sc-7309 | Santa Cruz | 1:1000 |
| Nedd8 | A13520 | ABclone | 1:1000 |
| β-actin | GB11001-100 | Servicebio | 1:1000 |

**Supplemental Table 3. Summary of transcriptome sequencing data.**

| Sample | Raw reads(bp) | Clean reads(bp) | Clean reads  Q20(%) | Clean reads  Q30(%) | Unique  Mapped(%) | Multiple  Mapped(%) | Total  Mapped(%) |
| --- | --- | --- | --- | --- | --- | --- | --- |
| DMSO-1 | 39786878 | 39058304 | 97.38% | 95.29% | 34620065 (93.87%) | 2260609 (6.13%) | 36880674 (94.42%) |
| DMSO-2 | 42419828 | 41541552 | 97.17% | 94.97% | 36918880 (94.16%) | 2291817 (5.84%) | 39210697 (94.39%) |
| DMSO-3 | 54101624 | 53205082 | 97.52% | 95.63% | 47247245 (93.96%) | 3038076 (6.04%) | 50285321 (94.51%) |
| MLN-1 | 46294102 | 45460594 | 97.42% | 95.33% | 40212660 (93.71%) | 2699820 (6.29%) | 42912480 (94.39%) |
| MLN-2 | 42970812 | 42064798 | 97.12% | 94.89% | 37358195 (93.88%) | 2436415 (6.12%) | 39794610 (94.60%) |
| MLN-3 | 41667840 | 40802892 | 97.12% | 94.9% | 36287268 (94.03%) | 2305256 (5.97%) | 38592524 (94.58%) |

Control group: DMSO1, DMSO2 and DMSO3; MLN4924 treatment group: MLN1, MLN2 and MLN3.

Methods: Upgrade version HISAT2 using TopHat2 (<http://ccb.jhu.edu/software/hisat2/index.shtml>).

The software compares the filtered Reads to the reference genome.

**Supplemental Table 4. Top 10 differential genes.**

| Gene Name | *P*-Value | Log2 FC | Regulation |
| --- | --- | --- | --- |
| MMP10 | 3.22E-10 | 4.102277317 | up |
| OTOGL | 0.019247261 | 4.047771243 | up |
| KLHL14 | 0.000102173 | -3.992652823 | down |
| CSF3 | 2.20E-18 | -3.969331817 | down |
| KATNAL2 | 0.000153925 | -3.857331077 | down |
| MAK | 0.009593688 | -3.835504212 | down |
| HKDC1 | 1.98E-114 | 3.745247336 | up |
| KCNJ16 | 5.79E-108 | 3.72668445 | up |
| GRM2 | 0.026020253 | -3.625926075 | down |
| NR1H4 | 4.56E-18 | 3.597505256 | down |

**Supplemental Table 5. Top 10 KEGG pathways.**

| Pathway ID | Pathway Name | *P*-Value |
| --- | --- | --- |
| ocu03320 | PPAR signaling pathway | 1.27E-05 |
| ocu04110 | Cell cycle | 7.88E-05 |
| ocu04657 | IL-17 signaling pathway | 9.11E-05 |
| ocu04270 | Vascular smooth muscle contraction | 0.000105333 |
| ocu00100 | Steroid biosynthesis | 0.000155983 |
| ocu04913 | Ovarian steroidogenesis | 0.000358004 |
| ocu04668 | TNF signaling pathway | 0.00044319 |
| ocu05200 | Pathways in cancer | 0.000556406 |
| ocu00533 | Glycosaminoglycan biosynthesis - keratan sulfate | 0.000997053 |
| ocu00480 | Glutathione metabolism | 0.0011305 |
